# Evaluation of the Mandibular Condyle Morphologic Relation before and after Orthognathic Surgery in Class II and III Malocclusion Patients Using Cone Beam Computed Tomography

**DOI:** 10.3390/biology11091353

**Published:** 2022-09-14

**Authors:** Raluca Roman, Oana Almășan, Mihaela Hedeșiu, Mihaela Băciuț, Simion Bran, Daiana Popa, Alina Ban, Cristian Dinu

**Affiliations:** 1 Department of Maxillofacial Surgery and Implantology, Iuliu Hațieganu University of Medicine and Pharmacy, 37 Iuliu Hossu Street, 400029 Cluj-Napoca, Romania; 2 Department of Prosthetic Dentistry and Dental Materials, Iuliu Hațieganu University of Medicine and Pharmacy, 32 Clinicilor Street, 400006 Cluj-Napoca, Romania

**Keywords:** orthognathic surgery, temporomandibular joint, mandibular condyle position, cone beam computed tomography

## Abstract

**Simple Summary:**

In individuals with severe malocclusions, orthognathic surgery seeks to rebalance the relationships between the jaws by providing a stable occlusion, a healthy muscle balance, and the functioning of the temporomandibular joint. Cone beam computed tomography may be used to determine the position of the mandibular condyle in the glenoid fossa. This study aimed to assess how the position of the mandibular condyle varies in class II and III malocclusions before and after bimaxillary orthognathic surgery. Before and after orthognathic surgery, 56 TMJs from 28 patients were studied. Following surgery, both class II and class III patients experienced changes in the anterior joint space, posterior joint space, condyle position, and condyle angle. The preliminary findings are promising for determining changes in condyle position and joint spaces that might guide oral and maxillofacial surgeons to address a debilitating clinical affliction.

**Abstract:**

This study aimed at evaluating the mandibular condyle position changes before and after bimaxillary orthognathic surgery in class II and III malocclusion patients. CBCT scans from patients who underwent bimaxillary orthognathic surgery were analyzed: Le Fort I osteotomy and bilateral sagittal split osteotomy (BSSO). Both condyles were independently assessed for their largest anterior and posterior joint spaces, smallest medial joint spaces, and condyle angles concerning the transverse line. In the sagittal plane, the minimum size of the anterior and posterior joint spaces was measured. In the coronal plane, the smallest medial joint space was measured. The position of the condyle within the glenoid fossa was determined before and after surgery. A total of 56 TMJs from 28 patients were studied. Following orthognathic surgery, the anterior and posterior space in class II increased. Postoperatively, the anterior joint space in class III decreased. In 42.85% of malocclusion class II patients and 57.14% of malocclusion class III patients, the pre-and post-surgical position of the condyle changed, the condyle was anteriorly positioned (42.85%) in class II patients and centrically positioned (71.4%) in class III patients. Significant changes in the joint space, condylar position, and condyle angle were found in the class II and class III subjects.

## 1. Introduction

Orthognathic surgery aims to rebalance the functions of the dentomaxillary apparatus by obtaining a stable occlusion, a proper muscle balance, and flawless physiological functioning of the temporomandibular joint (TMJ). The planning of the surgical procedure requires a thorough assessment of all of the anatomical structures involved and their intricate relations, with imaging playing a crucial part in this process.

Cone beam computed tomography (CBCT) is a three-dimensional imaging technique, mainly used for the head and neck area due to its cone-like radiation beam and low radiation dose [1]. Compared to computed tomography (CT), CBCT delivers lower radiation doses for patients, with the same or sometimes even higher accuracy of the image of the skeletal structure and the ability to process a large amount of information [2,3].

The diagnosis and examination of the TMJ are improved by multiplanar radiography imaging and three-dimensional reconstruction of the condyle and adjacent bone structures [4,5]. The condyle position in the glenoid fossa may be determined by CBCT, enabling the execution of very accurate condylar measurements and the qualitative assessment of the condyle surface by morphological evaluation [6].

The CBCT images of the articular space and the morphology of the condyle can highlight specific pathologies such as condylar abnormalities, ankylosis, and degenerative or rheumatoid disease, thus guiding the clinician to an appropriate treatment plan [4,7,8].

Even when not specifically targeting the TMJ, orthognathic surgery affects this region through muscle forces. Following orthognathic surgery, the mandibular muscles display a range of adaptive responses [9]. Individuals having bimaxillary orthognathic surgery may have a relapse and temporomandibular dysfunction as postoperative consequences [10]. Following orthognathic surgery, postoperative condylar movement may often produce recurrence or severe symptoms of temporomandibular joint disorders, therefore, various methods have been presented to reduce the dislocation, among them digital ones, or manual adjusting [11].

The functioning of the TMJ such as the amplitude of mandibular movements, the absence or presence of joint sounds, or the capacity to perform the maximum mouth opening may be utilized to assess the success of orthognathic surgery [12].

There are debates over post-operative changes at the TMJ level following orthognathic surgery. These controversies can be determined by the postoperative interval when the follow-up is conducted, and the method used to evaluate the position of the condyle. Several authors have reported post-operative changes. Shao et al. showed that the condyles’ positions changed after bimaxillary orthognathic surgery; after the procedures, the preoperative stress asymmetries of the left and right TMJs were still present [13]. Lee et al., on the other hand, agreed that evaluating the displacement of the mandibular condyle, the TMJ’s anterior, superior, or posterior joint spaces did not significantly alter after orthognathic surgery [14]. Francisco et al. showed that in class II patients, the altered condylar shape may be the result of BSSO with mandible advancing therapy, and condyle degradation might develop [15].

Throughout orthognathic surgery, bone fragment manipulation often leads to mandibular condyle structural alterations, however, the available literature on the studied issue is marked by a high degree of variability in terms of the study goal and methodology [16].

TMJ modifications may be caused by dysfunction of the masticatory system in patients with malocclusion. Individuals who undergo orthognathic surgery benefit from a repositioning of the mandibular condyle to an improved equilibrium state position during postoperative physiotherapy [17].

Individuals with class II malocclusion could have a difference in condyle location, osseous structure, and joint fossa morphology compared to those with class I malocclusion with substantial variations in joint space, condyle dimensions, and fossa morphology [18], although other authors established, using a CBCT comparison analysis, that there was no substantial variation among the class II/2 and class I orthodontic patients in terms of condyle sagittal placement [19]. In a study of the location and morphology, Lin et al. showed that the location and morphology of the mandibular condyle in patients with skeletal class II malocclusion, compared to class I, were different between the hypodivergent, normal, and hyperdivergent cases, individuals who had a high angle showed an instability of the TMJ, smaller diameters of the condyle, and significantly reduced the depth of the glenoid fossa [20]. Examining the features of the TMJ and adjacent elements across classes I, II, and III, Song et al. found that class III patients had a more proximally relocated condyle placement compared to class I subjects [21].

Few studies have evaluated the TMJ changes in malocclusion patients before and after orthognathic surgery. The anatomy of the temporomandibular joint and the position of the condyle are not directly aimed at in orthognathic surgery treatment [22]. However, since it significantly lessens pain and clicking, orthognathic surgery positively impacts TMJ dysfunction [23]. On the other hand, condylar remodeling following orthognathic surgery may occur, with condylar resorption being a possible outcome in certain patients, and additional elucidation of this phenomenon and improved characterization of its etiology may be feasible in the years ahead by means of using CBCT and cephalometric analysis [24]. Many variables, both before and after surgery, may contribute to a heightened state of stress by inducing inflammatory responses and biochemical sequences that may lead to physiological remodeling, and pathological remodeling can result from excessive physiological remodeling in condylar resorption or degenerative joint disease may occur when this process goes too far [25].

This study aimed to evaluate the mandibular condyle position changes before and after bimaxillary orthognathic surgery in class II and III malocclusion.

## 2. Materials and Methods

The present retrospective observational study analyzed twenty-eight CBCT examinations performed between 2016 and 2017 at the Maxillofacial Clinic in Cluj-Napoca, Romania. CBCT scans from patients who underwent bimaxillary orthognathic surgery were analyzed. The surgical procedure consisted of Le Fort I osteotomy and bilateral sagittal split osteotomy (BSSO).

Each patient underwent a close mouth CBCT examination (Promax 3D Max, Planmeca, Finland) before (T0) and at two days after the orthognathic surgery (T1) with the following protocol: field of view (FOV) 23 × 23 × 16 cm, 0.4 mm voxel size, exposure parameters ranging from 86 to 88 kilovoltage (kV), 6–8 milliamperage (mA), and 13.5 s (s) time of exposure. Each patient underwent the same surgical protocol and CBCT imaging. The CBCT scans were analyzed using variant 3.8.0 Planmeca Romexis software.

The inclusion criteria were skeletal class II or class III malocclusion, bimaxillary orthognathic surgery, and the existence of pre-and postsurgical CBCT scans obtained with the same equipment.

The exclusion criteria were patients with unilateral orthognathic surgery, ample TMJ pathology, absence of one examination (pre- or post-surgery), and CBCT examination that presented artifacts, either due to movement during the procedure or excessive metallic ones, thus preventing correct measurements.

The study obtained ethical approval, all procedures followed the ethical standards of the responsible committee on human experimentation (institutional and national), and the 1975 Declaration of Helsinki, as revised in 2008. The study was approved by the Iuliu Hațieganu University of Medicine and Pharmacy ethics committee (approval number 125.19.05.2017). All participants in the study were informed and signed consent forms.

The measurements were performed on the oblique sagittal and coronal reformatted images to eliminate any possible interference in the objectivity of the measurements between the two examinations.

The following variables were evaluated individually for both condyles in each class of malocclusion (II and III) before and after surgery: the largest anterior and posterior joint spaces, the smallest medial joint space, and the condyle angle in relation to the transverse line.

**Figure 1:**
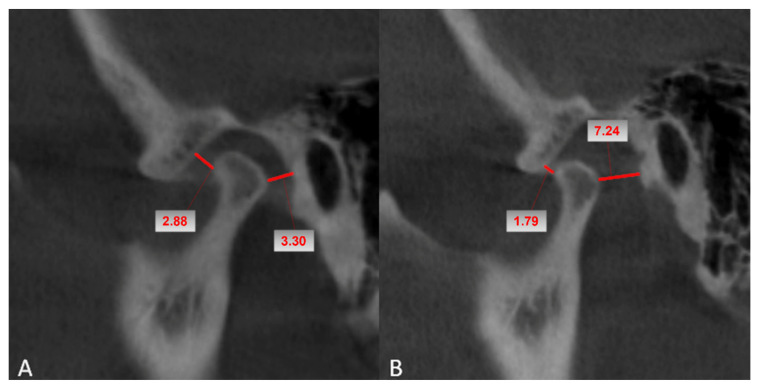
CBCT examination with a closed mouth. Sagittal plane. TMJ anterior and posterior space measurements: (**A**) preoperative, (**B**) postoperative.

In the sagittal plane, the minimum size of the anterior and posterior TMJ spaces, defined as the distance between the most anterior point of the condyle, respectively, and the cortex of the glenoid cavity, were measured (Figure 1).

The method described by Pullinger et al., which defines the condyle position (PC) by utilizing the smallest posterior space (P) and the largest anterior distance (A) on the sagittal view, was used to classify the position of the condyle inside the glenoid fossa [26,27]. Accordingly, we calculated the condyle position using the formula PC (%) = [(P − A) × 100]/(P + A). Based on the measurements of the anterior and posterior joint spaces on the sagittal plane, we determined the position of the condyle (centric, anterior, or posterior) in the glenoid fossa: centric position [PC between (−)12% and (+)12%], posterior position [PC below (−)12%], and anterior position [PC above (+)12%] [28].

**Figure 2:**
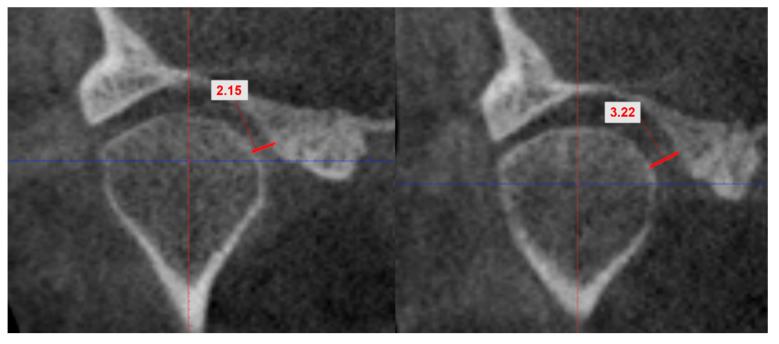
CBCT coronal section. The measurements of the minimal length of the medial joint space.

In the coronal plane, the smallest medial joint space was measured as the distance between the most medial point of the condyle and the medial wall of the glenoid cavity (Figure 2).

**Figure 3:**
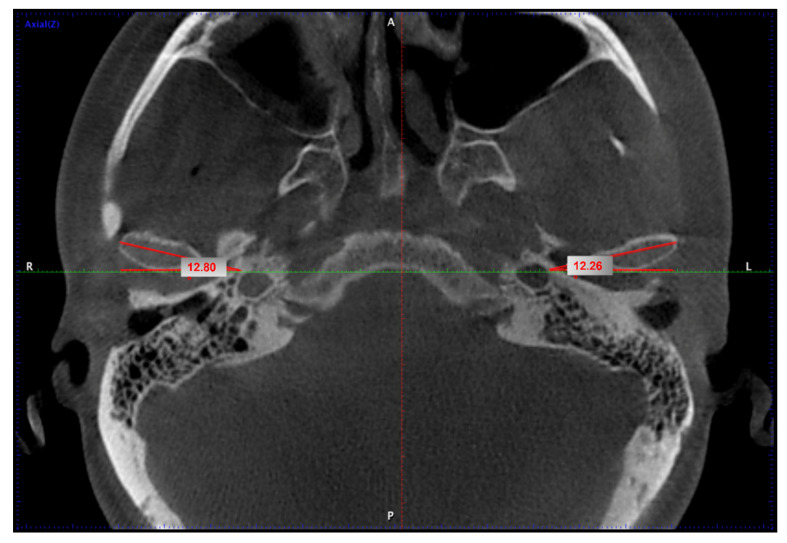
CBCT axial section. The measurements of the condyle angle performed in its largest diameter related to the transversal plane.

The angle between the maximal diameter of the condyle in the axial plane and the transverse line was used to calculate the rotation of the condyle in the axial plane (Figure 3).

Statistical analysis was performed using IBM SPSS Statistics 22 (Armonk, NY, USA). The Shapiro–Wilk test and the Q–Q plot distribution were used to assess the normality of the data distribution. Standard deviation (SD), median, interquartile range (IQR), minimum and maximum values, and mean were used to characterize the quantitative variables. The Wilcoxon Signed-rank test at a significance level of 0.05 for evaluating the differences between the pre-and post-surgery variables in the groups and the Spearman’s correlation coefficient for evaluating the strength and direction of association between variables were used.

## 3. Results

A total of 56 TMJs from 28 patients were studied before and after orthognathic surgery. Subjects were grouped according to the skeletal malocclusion into class II and class III subjects with an equal number of 28 patients encountered in each class. The mean age in malocclusion class II was 26.85 years (range 16–44 years; ±6.54 SD), with twelve females (85.7%) and two males (14.45%), while the mean age in class III was 26.64 years (range 17–45 years; ±6.87 SD), with seven males (50%) and seven females (50%).

Table 1, Table 2 and Table 3 describe the TMJ space values on pre-surgical (T0) and post-surgical (T1) CBCT evaluations. TMJ space intra- and inter-class comparisons revealed significant post-operative changes in the anterior and posterior joint space dimensions in class II malocclusion patients (*p* < 0.05). The overall measurements demonstrated that the anterior and the posterior space increased following orthognathic surgery (T1) compared to the pre-operative space (T0) and the mean difference was 0.61 ± SD 0.91 mm and 1.16 ± SD 1.24 mm, respectively.

In class III malocclusion patients, the anterior TMJ space decreased post-operatively (mean differences were 0.37 ± SD 0.40 mm) and the posterior joint space increased (mean differences were 0.43 ± SD 0.63 mm) with no statistically significant differences between the pre-surgical and post-surgical values.

The position of the condyle concerning the medial joint cavity wall showed, in class II, values between 1.60 to 6 mm pre-surgery, with changes after surgery to 1.60 to 5.60 mm, and a mean value of 0.17 mm closer to the medial wall. In class III, the position pre-surgery had a mean value of 2.05 mm for the right joint and 2.00 mm for the left joint, and minimally changed to a mean value of 0.66 mm for the right joint and 0.40 mm for the left one (Table 4). Testing the post-operative changes in medial joint space, significant changes were found only in class III malocclusion patients with a size difference in the mean value of 0.67 ± SD 0.79 mm. 

There was a change in the pre-and post-surgical position of the condyle in 42.85% of the malocclusion class II patients and 57.14% of the malocclusion class III patients after evaluating the condylar position in the glenoid fossa (Table 5). Following orthognathic surgery, the mandibular condyle was most frequently anteriorly positioned (42.85%) in the class II malocclusion patients, and centrically positioned (71.4%) in the class III malocclusion patients.

The condyle angle values in class II for the right condyle ranged between 12.76° and 44.19° pre-surgery and 9.45° to 41.63° post-surgery in the same class, whereas class III presented values of the right condyle angulation between 6.58° and 28.71° pre-surgery and 8.02° to 28.40° post-surgery, respectively. For the left condyle, in class II, there were values between 14.62° and 45.00° pre-surgery and 4.88 to 41.31 post-surgery. In class III, the left condyle had an angulation between 7.62° and 27.03° pre-surgery and between 6.45° and 25.51° post-surgery. Class II malocclusion showed, between pre- and post-surgery, a decrease in the mean angle values of 3.98° on the right side and 5.32° on the left side. Class III malocclusion presented negative angle mean values of the difference between the two examination moments, with −2.52° on the right side and −1.13° on the left side.

## 4. Discussion

Before any orthognathic surgical procedure, regardless of whether it is a class II or III malocclusion, it is essential to evaluate the correlation between cephalometric variables and the temporomandibular joint and to define the skeletal pattern [29]. CBCT obtained 3D representations can be used to describe the intrinsic variability of the mandible in healthy individuals [30]. In this study, we explored the relationships between the TMJ components, along with the mandibular condyle and the temporal bone, based on the anterior and posterior joint spaces, the medial joint space, and the condyle angle concerning the transverse line using CBCT. On the other hand, CBCT may be employed to create three-dimensional simulations utilizing mandible characteristics [31].

Our investigation found post-operative changes in the position of the condyle in the glenoid cavity as well as changes in the joint space and the axis of rotation of the condyle regarding the transverse plane of the skull in both the class II and class III patients. Overall, it appears that following surgery, the condyle may appear in a comparable position concerning the medial wall of the cavity in both groups. This outcome was seen in one-third of the joints in our study.

Vale et al., aiming to analyze the effectiveness of CBCT in evaluating the condylar position, angulation, and intercondylar distance and to assess the changes in these parameters before and after bimaxillary surgery in class II patients, showed that the surgery did not significantly change the mandibular condyles. However, they tended to migrate inferiorly and posteriorly [32]. These findings are equivalent to the current study’s findings for patients with class II malocclusion.

Da Silva et al., investigating changes in the condylar volume and joint spaces during orthognathic surgery in class II patients showed that in the follow-up examinations performed six months post-surgery, the superior and medial joint spaces were significantly decreased [33]. When the changes in the joint spaces at the level of the right and left condyle were correlated, the posterior space showed a moderate correlation. In contrast, the anterior space showed a weak correlation. Our study showed a strong correlation between the anterior joint space of the right and left condyle. We also found a strong correlation between the minimal length of the right and left medial joint space.

Before any operative surgery, it is recommended to evaluate the facial disproportion and the condition of the TMJ, since it is known that asymmetrical TMDs are related to changes in anthropometric measures, as changes in some cephalometric parameters might become an important component in determining the existence of TMDs [34]. The mandibular plane and skeletal class were related to the sagittal position of the TMJ, with a broader mandibular angle and more anterior condyle position in class II individuals and a smaller mandibular angle and more posterior condyle position in class III patients [35]. The angle of the condyle showed a prominent more oblique position toward the transversal plane of the skull. After surgery in the class II patients, the condyle angulation decreased for both the right and left condyles in over 80% compared to the class III cases, where the angle showed increasing values in over two-thirds of the patients. These results were compatible with repositioning the anterior part of the mandibular arch after BSSO, and the variability in the values was produced by a different range of advancement for class II and posterior repositioning for class III.

Long-term TMJ stability after orthognathic surgery was studied by Kim et al., who assessed how the condyle’s position had changed up to a year following orthognathic surgery in patients with Class III malocclusion, and showed that at three months, the condyles showed anterior displacement; by six months, they had settled back into a more distal position [36]. After three months, the condylar rotated forward and remained stable in the sagittal view, tending to shift in a specific direction, thus affecting postsurgical relapse for up to six months following surgery; after that, they remained steady [36]. The condyle position changed after surgery in the case of the current study. Condylar alterations and joint stability were examined after six months and after 18 months in the research by Kim et al. on class III patients who had bimaxillary surgery, they noted that after six months, the condyle posture altered to a centric position; however, at the 18-month assessment, the condyle’s position had moved to a more anterior position than it had been seen previously, the stability being unaffected by those changes [37]. The increased tension in the muscle tissue may be what enabled the condyle in the glenoid fossa to revert to its pre-surgery state [38]. The temporomandibular joint can adjust to the changed skeletal position generated by the surgical treatment, with the degree of the condyle position’s recurrence depending on the tissues’ physiological adaptability. The types of fixation employed, the management of the proximal segment during surgery, and the surgeon’s expertise affect the stability of the condyle’s location in the glenoid fossa over time [37].

In class II patients with orthognathic surgery and botulinum toxin injection, significant changes in the anterior belly of the digastric muscle have been noted including lengthening and thinning of the masticatory muscles [26]. This effect might positively affect the postoperative TMJ stability in orthognathic patients.

Accurate maxillary positioning is essential in bimaxillary surgery [39]. After bone healing has taken place, the removal of the plates used in orthognathic surgery is indicated [40]. This should not determine essential changes in the TMJ other than the progression of the adaptive muscle process, since the new morphology of the arch is already consistent, mainly if optimal and stable occlusion relations are produced. The technique in which orthognathic surgery is performed can impact TMJ stability, and as a consequence, the position of the condyles in the glenoid cavity.

The study’s strengths are the comparison of both malocclusion classes, II and III, concerning the condyle position and angulation changes, pre- and post-orthognathic surgery. One potential limitation of our study is that the range of advancement for class II patients following BSSO was not measured and correlated with the degree of condyle relation changes. Another limitation of the study is the relatively reduced number of subjects per class and the early second examination moment, which may result in modification of the data related to the functional relation of the condyle within the glenoid cavity over time.

## 5. Conclusions

Evaluation of the morphologic relationship between the mandibular condyle before and after orthognathic surgery is a subject of intense scientific interest. Condylar position changes were present in patients with class II and class III malocclusion who had bimaxillary orthognathic surgery in our study. Significant changes in joint space, condylar position, and condyle angle concerning the transversal plane of the skull were seen one week after surgery. These findings highlight the importance of long-term follow up evaluations of TMJ stability in orthognathic surgery patients.

## Figures and Tables

**Table 1:** The characteristics of the studied variables in class II malocclusion patients (*n* = 28).

Variable Class II	Mean	SD	Median	IQR	Min.	Max.	*p*-Value Pre- and Post-Surgery (Wilcoxon Signed-Rank Test)	Spearman’s Rho Correlation Coefficient Pre- and Post-Surgery and *p*-Value
Anterior joint space T0 (mm)	2.45	0.62	2.40	0.70	1.60	4.80	0.028 *	0.477 * 0.010 *
Anterior joint space T1 (mm)	2.87	1.10	2.40	1.40	2.00	6.80		
Posterior joint space T0 (mm)	2.75	1.53	2.40	1.10	1.20	8.00	0.011 *	0.639 ** 0.0001 **
Posterior joint T1 (mm)	3.49	1.69	3.30	2.40	1.60	7.60		
Medial joint space’s minimal length T0 (mm)	2.98	1.10	2.80	2.00	1.60	6.00	0.410	0.633 ** 0.0001 **
Medial joint space’s minimal length T1 (mm)	2.91	1.29	2.00	2.30	1.60	5.60		
Condyle angle T0 (degrees)	27.87	10.48	26.19	21.21	12.76	45.00	0.0001 **	0.924 ** 0.0001 **
Condyle angle T1 (degrees)	23.21	9.98	25.65	17.54	4.88	41.63		

T0—pre-surgery, T1—post-surgery, SD—standard deviation, IQR—interquartile range, Min—minimum; Max—maximum; * *p* is significant at the 0.05 level; ** *p* is significant at the 0.001 level.

**Table 2:** Characteristics of the studied variables in the class III malocclusion patients (*n* = 28).

Variable Class III	Mean	SD	Median	IQR	Min.	Max.	*p*-Value Pre- and Post-Surgery (Wilcoxon Signed-Rank Test)	Spearman’s Rho Correlation Coefficient Pre- and Post-Surgery and *p*-Value
Anterior joint space T0 (mm)	2.15	0.63	2.00	0.80	1.20	4.00	0.139	0.737 ** 0.0001 **
Anterior joint space T1 (mm)	2.0	0.62	1.80	0.80	1.20	4.40		
Posterior joint space T0 (mm)	2.01	0.44	2.00	0.80	1.60	3.20	0.531	0.466 * 0.012 *
Posterior joint T1 (mm)	2.16	0.80	2.00	0.80	1.20	5.60		
Medial joint space’s minimal length T0 (mm)	2.02	0.72	1.60	1.10	1.20	3.60	0.006 *	0.608 ** 0.001 **
Medial joint space’s minimal length T1 (mm)	2.55	1.11	2.40	1.90	1.20	5.20		
Condyle angle T0 (degrees)	16.34	5.91	15.70	7.69	6.58	28.71	0.007 *	0.838 ** 0.0001 **
Condyle angle T1 (degrees)	18.17	5.52	17.44	7.66	6.45	28.40		

T0—pre-surgery, T1—post-surgery, SD—standard deviation, IQR—interquartile range, Min—minimum; Max—maximum, * *p* is significant at the 0.05 level; ** *p* is significant at the 0.001 level.

**Table 3:** The change values between T0–T1 moments (*n* = 28, absolute value).

Class II	Mean	SD	Median	IQR	Min.	Max.	Class III	Mean	SD	Median	IQR	Min.	Max.
Anterior joint space (mm)	0.61	0.91	0.40	0.80	0	4.40	Anterior joint space (mm)	0.37	0.40	0.40	0.40	0	1.60
Posterior joint space (mm)	1.16	1.24	0.80	1.50	0	4.80	Posterior joint space (mm)	0.43	0.63	0.40	0.40	0	3.20
Medial joint space’s minimal length (mm)	0.75	0.77	0.40	1.00	0	2.80	Medial joint space’s minimal length (mm)	0.67	0.79	0.40	1.20	0	2.80
Condyle angle (degrees)	4.96	3.80	4.03	6.36	0.37	13.14	Condyle angle (degrees)	2.58	2.20	2.29	3.22	0.07	8.60

T0—pre-surgery, T1—post-surgery, SD—standard deviation, IQR—interquartile range, Min—minimum; Max—maximum.

**Table 4:** The characteristics of the studied variables in class II and III, left and right joints.

Variable Class II (*n* = 14 in Each Side: Right and Left)	Mean	SD	Median	IQR	Min.	Max.	*p*-Value (Wilcoxon Signed-Rank Test)	Spearman’s Rho Correlation Coefficient and *p*-Value
Right anterior joint space T0 (mm)	2.62	0.82	2.40	0.90	1.60	4.80	0.226	0.705 * (*p* = 0.005)
Right anterior joint space T1 (mm)	2.82	0.92	4.40	1.60	2.00	5.20		
Right posterior joint space T0 (mm)	2.91	1.81	2.40	1.10	1.20	8.00	0.074	−0.155 (*p* = 0.59)
Right posterior joint T1 (mm)	3.85	1.85	3.60	2.90	2.00	7.60		
Right medial joint space’s minimal length T0 (mm)	3.25	1.24	3.20	1.80	1.60	6.00	0.474	0.62 * (*p* = 0.01)
Right medial joint space’s minimal length T1 (mm)	3.08	1.41	2.80	2.20	1.60	5.60		
Right condyle angle T0 (degrees)	27.85	10.38	25.36	19.17	12.76	44.19	0.004	0.91 ** (*p* < 0.001)
Right condyle angle T1 (degrees)	23.87	10.07	25.65	18.52	9.45	41.63		
Left anterior joint space T0 (mm)	2.28	0.24	2.40	0.40	2.00	2.80	0.076	0.05 (*p* = 0.84)
Left anterior joint space T1 (mm)	2.91	1.29	2.40	1.10	2.00	6.80		
Left posterior joint space T0 (mm)	2.60	1.23	2.20	1.50	1.20	5.20	0.058	0.74 * (*p* = 0.002)
Left posterior joint space T1 (mm)	3.12	1.49	3.00	2.50	1.60	6.40		
Left medial joint space’s minimal length T0 (mm)	2.71	0.90	2.60	1.40	1.60	4.40	0.765	0.60 * (*p* = 0.02)
Left medial joint space’s minimal length T1 (mm)	2.74	1.19	2.00	2.40	1.60	4.80		
Left condyle angle T0 (degrees)	27.88	10.97	26.57	23.22	14.62	45.00	0.002	0.90 ** (*p* < 0.0001)
Left condyle angle T1 (degrees)	22.56	10.23	22.72	16.25	4.88	41.31		
Variable Class III (*n* = 14 in each side: right and left)								
Right anterior joint space T0 (mm)	2.20	0.53	2.20	0.60	1.20	3.20	0.143	0.69 * (*p* = 0.006)
Right anterior joint space T1 (mm)	2.01	0.41	2.00	0.80	1.60	2.80		
Right posterior joint space T0 (mm)	2.05	0.43	2.00	0.80	1.60	3.20	0.711	0.29 (*p* = 0.31)
Right posterior joint T1 (mm)	2.12	0.45	2.00	0.90	1.60	2.80		
Right medial joint space’s minimal length T0 (mm)	2.05	0.75	1.80	1.20	1.20	3.60	0.021	0.63 * (*p* = 0.01)
Right medial joint space’s minimal length T1 (mm)	2.71	1.19	2.80	2.10	1.20	5.20		
Right condyle angle T0 (degrees)	17.08	6.13	15.98	7.46	6.58	28.71	0.016	0.72 * (*p* = 0.003)
Right condyle angle T1 (degrees)	19.60	5.28	19.07	6.56	8.02	28.40		
Left anterior joint space T0 (mm)	2.11	0.74	2.00	0.80	1.20	4.00	0.473	0.77 ** (*p* = 0.001)
Left anterior joint space T1 (mm)	2.00	0.80	1.60	0.50	1.20	4.40		
Left posterior joint space T0 (mm)	1.97	0.45	1.80	0.80	1.60	2.80	0.618	0.54 * (*p* = 0.04)
Left posterior joint space T1 (mm)	2.20	1.06	2.00	0.80	1.20	5.60		
Left medial joint space’s minimal length T0 (mm)	2.00	0.73	1.60	0.90	1.20	3.60	0.127	0.61 * (*p* = 0.01)
Left medial joint space’s minimal length T1 (mm)	2.40	1.04	2.20	1.80	1.20	4.40		
Left condyle angle T0 (degrees)	15.61	5.82	15.04	7.56	7.65	27.03	0.198	0.86 ** (*p* < 0.0001)
Left condyle angle T1 (degrees)	16.74	5.56	15.99	7.74	6.45	25.51		

T0—pre-surgery, T1—post-surgery, SD—standard deviation, IQR—interquartile range, Min—minimum; Max—maximum, * *p* is significant at the 0.05 level; ** *p* is significant at the 0.001 level.

**Table 5:** The condylar position pre- and post-surgery according to Pullinger et al.’s formula [26,27] (*n* = 28).

	T0			T1		
	Anterior	Centric	Posterior	Anterior	Centric	Posterior
Right class II	4	5	5	6	6	2
Right class III	1	9	4	3	11	0
Left class II	3	8	3	6	3	5
Left class III	2	9	3	4	9	1

T0—pre-surgery, T1—post-surgery.

## Data Availability

The data presented in this study are available from the first author upon reasonable request.
